# Impact of comorbidity burden on mortality in patients with COVID-19 using the Korean health insurance database

**DOI:** 10.1038/s41598-021-85813-2

**Published:** 2021-03-18

**Authors:** Soo Ick Cho, Susie Yoon, Ho-Jin Lee

**Affiliations:** 1 Department of Dermatology, Seoul National University Hospital, Seoul, Republic of Korea; 2 Department of Anesthesiology and Pain Medicine, Seoul National University Hospital, Seoul, Republic of Korea; 3 Department of Anesthesiology and Pain Medicine, Seoul National University College of Medicine, Seoul, Republic of Korea

**Keywords:** Infectious diseases, Respiratory tract diseases

## Abstract

We aimed to investigate the impact of comorbidity burden on mortality in patients with coronavirus disease (COVID-19). We analyzed the COVID-19 data from the nationwide health insurance claims of South Korea. Data on demographic characteristics, comorbidities, and mortality records of patients with COVID-19 were extracted from the database. The odds ratios of mortality according to comorbidities in these patients with and without adjustment for age and sex were calculated. The predictive value of the original Charlson comorbidity index (CCI) and the age-adjusted CCI (ACCI) for mortality in these patients were investigated using the receiver operating characteristic (ROC) curve analysis. Among 7590 patients, 227 (3.0%) had died. After age and sex adjustment, hypertension, diabetes mellitus, congestive heart failure, dementia, chronic pulmonary disease, liver disease, renal disease, and cancer were significant risk factors for mortality. The ROC curve analysis showed that an ACCI threshold > 3.5 yielded the best cut-off point for predicting mortality (area under the ROC 0.92; 95% confidence interval 0.91–0.94). Our study revealed multiple risk factors for mortality in patients with COVID-19. The high predictive power of the ACCI for mortality in our results can support the importance of old age and comorbidities in the severity of COVID-19.

## Introduction

The coronavirus disease (COVID-19), an infectious disease caused by the severe acute respiratory syndrome coronavirus 2 (SARS-CoV-2), was first reported in Wuhan, Hubei, China at the end of December 2019^1^ and has rapidly spread worldwide. On March 11, 2020, the World Health Organization (WHO) declared it a pandemic^2^. As of May 30, 2020, the global death toll from COVID-19 had exceeded 340,000 according to WHO. Nevertheless, clinical data to guide health care professionals or policy makers in their decision-making is still scarce. Against this backdrop, the government of the Republic of Korea decided to share the COVID-19 nationwide claims data for global research^3^.

Detection of risk factors for mortality is an important component of the strategies for managing COVID-19. This information is all the more important at a time when the demand for critical care is surging and the resources for healthcare are limited^4,5^. A recent case series suggested old age and comorbidities as risk factors for severity of COVID-19^6–8^. However, information on how the combination of these risk factors affects the severity of COVID-19 is rare. We also thought that a simple but predictable model is needed for effective health care resource allocation for this public health emergency.

The Charlson comorbidity index (CCI) has been validated for predicting mortality in patients^9^. CCI quantifies the risk of mortality associated with 19 weighted comorbidities, including congestive heart failure, cerebrovascular disease, chronic pulmonary disease, and diabetes, all of which have been reported as prognostic factors of poor outcome in patients with COVID-19^9^. Recently, the CCI score has been reported to be associated with the mortality and disease severity of COVID-19^10^. In addition to weighting comorbidities, Charlson and colleagues also proposed the age-adjusted CCI (ACCI) by adding 1 point for every decade after 40 years of age^11^. Considering the importance of aging and comorbidities in the severity of COVID-19, we expect that ACCI could predict the mortality rate for COVID-19.

In this study, we investigated the age- and sex-adjusted odds ratio (OR) of mortality for each comorbidity and the predictive value of mortality provided by the ACCI for patients with COVID-19 from a nationwide claims database in South Korea. Our results provide valuable information for the identification of patients at high risk of critical illness and might need early intensive care.

## Results

**Figure 1:**
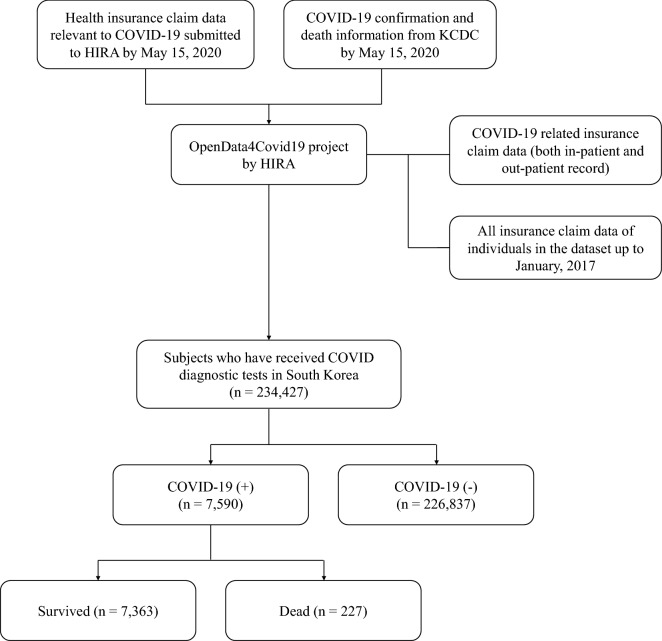
Summary of the dataset of our data. *HIRA* Health Insurance Review & Assessment Service, *KCDC* Korea Centers for Disease Control and Prevention.

In this study, 234,427 (male/female [M/F]: 111,947/122,480) patients had received the diagnostic test for COVID-19, and consequently, 7590 of them (3.2%, M/F: 3095/4495) had been confirmed with COVID-19 in South Korea (Fig. 1). Of this, a total of 7157 (94.3%) patients were admitted to the hospital for COVID-19. Among the hospitalized patients, 216 (3.0%) were admitted to the intensive care unit, 127 (1.8%) received mechanical ventilation, and 21 (0.3%) received extracorporeal membrane oxygenation (ECMO). A total of 227 (3.0%, M/F: 121/106) had died (in-hospital death: 218, out-of-hospital death: 9).

The associations between the patients’ demographics, clinical characteristics, and death in the all-patients cohort and Daegu–Gyeongbuk region cohort, are shown in Table 1. In the all-patient cohort, the most common comorbidity was hypertension (n = 1463, 19.3%), followed by chronic pulmonary disease (n = 958, 12.6%) and diabetes mellitus (n = 907, 11.9%). The non-surviving patients were significantly older, predominantly male, covered by medical aid, lived in the Daegu–Gyeongbuk region, and were more likely to have a history of all comorbidities. These characteristics were similar for the Daegu–Gyeongbuk cohort. The patients’ demographics, clinical characteristics, and death by sex are shown in Supplementary Table S1.

**Table 1:** Baseline characteristics and comorbidity of patients with coronavirus disease (COVID-19). Data are presented as number (%).

	Patients with COVID-19 (n = 7590)			Patients with COVID-19 in the Daegu–Gyeongbuk region (n = 4234)		
	Survivor	Non-survivor	P-value	Survivor	Non-survivor	P-value
Total number of patients	7363 (97.0)	227 (3.0)		4033 (95.3)	201 (4.7)	
**Sex**			< 0.001			< 0.001
Male	2974 (40.4)	121 (53.3)		1600 (39.7)	107 (53.2)	
Female	4389 (59.6)	106 (46.7)		2433 (60.3)	94 (46.8)	
**Age, y**	45 ± 19	77 ± 11	< 0.001	49 ± 19	77 ± 11	< 0.001
≤ 9	82 (1.1)	0 (0.0)		37 (0.9)	0 (0.0)	
10–19	346 (4.7)	0 (0.0)		132 (3.3)	0 (0.0)	
20–29	1855 (25.2)	0 (0.0)		809 (20.1)	0 (0.0)	
30–39	774 (10.5)	2 (0.9)		366 (9.1)	1 (0.5)	
40–49	1002 (13.6)	1 (0.4)		511 (12.7)	1 (0.5)	
50–59	1489 (20.2)	14 (6.2)		873 (21.6)	13 (6.5)	
60–69	1025 (13.9)	36 (15.9)		706 (17.5)	31 (15.4)	
70–79	523 (7.1)	67 (29.5)		386 (9.6)	58 (28.9)	
≥ 80	267 (3.6)	107 (47.1)		213 (5.3)	97 (48.3)	
**Insurance type**			< 0.001			< 0.001
Health insurance	6773 (92.0)	185 (81.5)		3612 (89.6)	162 (80.6)	
Medical aid	590 (8.0)	42 (18.5)		421 (10.4)	39 (19.4)	
**Cities and province**			< 0.001			
Daegu–Gyeongbuk region	4033 (54.8)	201 (88.5)				
Other cities and provinces	3330 (45.2)	26 (11.5)				
**Comorbidity**						
Hypertension	1307 (17.8)	156 (68.7)	< 0.001	919 (22.8)	135 (67.2)	< 0.001
Diabetes mellitus	799 (10.9)	108 (47.6)	< 0.001	578 (14.3)	100 (49.8)	< 0.001
Congestive heart failure	168 (2.3)	44 (19.4)	< 0.001	127 (3.1)	40 (19.9)	< 0.001
Cerebrovascular disease	339 (4.6)	53 (23.3)	< 0.001	281 (7.0)	48 (23.9)	< 0.001
Liver disease	566 (7.7)	42 (18.5)	< 0.001	390 (9.7)	39 (19.4)	< 0.001
Renal disease	44 (0.6)	15 (6.6)	< 0.001	36 (0.9)	13 (6.5)	< 0.001
Chronic pulmonary disease	875 (11.9)	83 (36.6)	< 0.001	569 (14.1)	71 (35.3)	< 0.001
Cancer	236 (3.2)	27 (11.9)	< 0.001	156 (3.9)	24 (11.9)	< 0.001
Charlson comorbidity index (CCI)	0 (0–1)	3 (2–4)	< 0.001	0 (0–1)	3 (2–4)	< 0.001
Age-adjusted CCI	1 (0–2)	6 (4–8)	< 0.001	1 (0–3)	6 (5–8)	< 0.001

**Figure 2:**
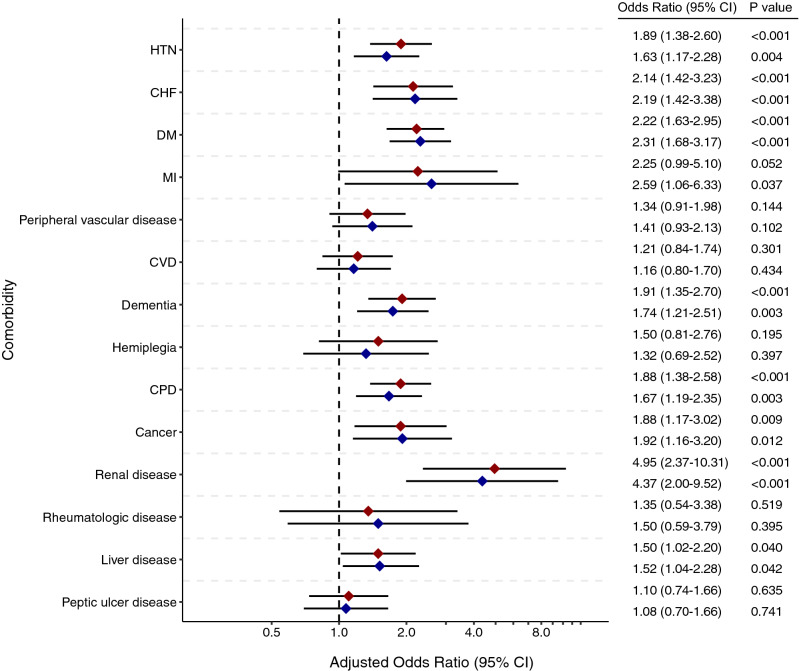
Age- and sex-adjusted odds ratios (95% confidence interval [CI]) of mortality according to the comorbidity of the all-patient (red) and Daegu–Gyeongbuk cohorts (blue) with coronavirus disease (COVID-19). *CHF* chronic heart failure, *CPD* chronic pulmonary disease, *CVD* cerebrovascular disease, *DM* diabetes mellitus, *HTN* hypertension, *MI* myocardial infarction. R: A language and environment for statistical computing. R Foundation for Statistical Computing, Vienna, Austria. https://www.R-project.org/.

Figure 2 shows the odds ratios (ORs) of mortality by comorbidities with 95% confidence intervals (CIs), with age and sex adjustment in the all-patient and the Daegu–Gyeongbuk cohorts. After adjustment, hypertension (OR 1.89; 95% CI 1.38–2.60), diabetes (OR 2.22; 95% CI 1.63–2.95), congestive heart failure (OR 2.14; 95% CI 1.42–3.23), dementia (OR 1.91; 95% CI 1.35–2.70), chronic pulmonary disease (OR 1.88; 95% CI 1.38–2.58), liver disease (OR 1.50; 95% CI 1.02–2.20), renal disease (OR 4.95; 95% CI 2.37–10.31), and cancer (OR 1.88; 95% CI 1.17–3.02) were significant risk factors for mortality in patients with COVID-19 in the all-patient cohort. A similar result was observed in the Daegu–Gyeongbuk cohort. The significant risk factors in both male and female patients were hypertension, diabetes, and chronic pulmonary disease (Supplementary Fig. S1). In addition, the CCI score was significantly associated with mortality in patients with COVID-19 (Supplementary Table S2).

**Figure 3:**
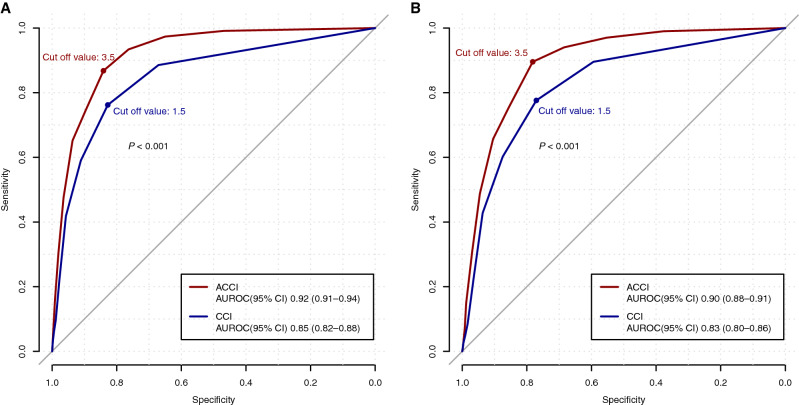
The area under the receiver operative characteristic curves of the Charlson comorbidity index (CCI) for predicting the mortality of all the patients (**A**) and patients living in the Daegu–Gyeongbuk region (**B**) with COVID-19. The blue and red lines represent the unadjusted and age-adjusted CCI, respectively. *ACCI* age-adjusted Charlson comorbidity index, *AUROC* area under the receiver operating characteristic, *CI* confidence interval. R: A language and environment for statistical computing. R Foundation for Statistical Computing, Vienna, Austria. https://www.R-project.org/.

In the all-patient cohort, the receiver operating characteristic (ROC) curve analysis showed that an ACCI threshold > 3.5 yielded the best cut-off point for predicting mortality (area under the ROC (AUROC); 0.92; 95% CI 0.91–0.94) with a corresponding sensitivity of 86.8%, specificity of 84.1%, positive predictive value (PPV) of 14.4%, and negative predictive value (NPV) of 99.5% (Table 2). The predicting performance of the ACCI was superior to that of the original CCI threshold > 1.5 (*P* < 0.001) (Fig. 3). In the Daegu–Gyeongbuk cohort, an ACCI threshold > 3.5 also yielded the best cut-off point for predicting mortality (AUROC 0.90; 95% CI 0.88–0.91), with a corresponding sensitivity of 89.5%, specificity of 78.2%, PPV of 17.0%, and an NPV of 99.3%. The predicting performance of the ACCI in the Daegu–Gyeongbuk cohort was also superior to that of the original CCI threshold > 1.5 (*P* < 0.001). The predicting performances of the CCI and ACCI in mortality among patients with COVID-19, according to sex, are shown in Supplementary Table S3 and Supplementary Fig. S2. In male patients, an ACCI threshold > 2.5 yielded the best cut-off point for predicting mortality.

**Table 2:** Sensitivity and specificity of the Charlson comorbidity index (CCI) in predicting mortality among patients with coronavirus disease (COVID-19).

	Sensitivity	Specificity	Youden index	PPV	NPV
**All patients with COVID-19**					
**Age-adjusted**					
CCI ≥ 3	0.934	0.763	1.697	0.108	0.997
CCI ≥ 4	0.868	0.841	1.709	0.144	0.995
CCI ≥ 5	0.744	0.897	1.641	0.182	0.991
**Unadjusted**					
CCI ≥ 1	0.885	0.671	1.556	0.077	0.995
CCI ≥ 2	0.762	0.828	1.590	0.120	0.991
CCI ≥ 3	0.590	0.911	1.501	0.170	0.986
**Patients with COVID-19 in the Daegu–Gyeongbuk region**					
**Age-adjusted**					
CCI ≥ 3	0.940	0.684	1.625	0.129	0.996
CCI ≥ 4	0.895	0.782	1.677	0.170	0.993
CCI ≥ 5	0.756	0.855	1.611	0.206	0.986
**Unadjusted**					
CCI ≥ 1	0.896	0.594	1.489	0.099	0.991
CCI ≥ 2	0.776	0.771	1.547	0.144	0.986
CCI ≥ 3	0.602	0.875	1.477	0.193	0.978

*PPV* positive predictive value, *NPV* negative predictive value.

**Figure 4:**
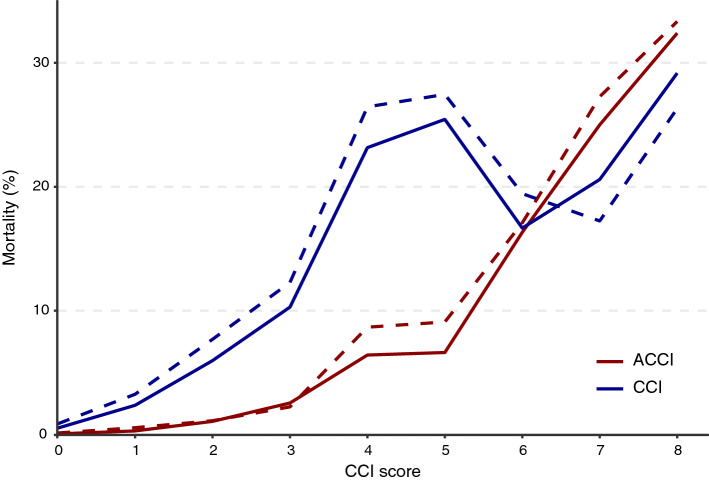
Mortality rate according to the Charlson comorbidity index (CCI) and age-adjusted CCI (ACCI) in the all-patient (solid line) and Daegu–Gyeongbuk cohorts (dotted line).

Figure 4 shows the mortality rate according to the CCI and ACCI in the all-patient and Daegu–Gyeongbuk cohorts. The mortality rate showed an increasing trend with the ACCI. The distributions of the CCI and ACCI in the all-patient cohort and Daegu–Gyeongbuk cohorts are shown in Supplementary Table S4. In the all-patient cohort, for those with an ACCI score of 4 or more, the mortality rate was 14.4% (197/1367), and for those with less than 4 points, the mortality rate was 0.5% (30/6223). In the Daegu–Gyeongbuk cohort, for those with an ACCI score of 4 or more, the mortality rate was 17.0% (180/1060), and for those with less than 4 points, the mortality rate was 0.7% (21/3174). Among the 433 non-hospitalized patients, a total of 9 patients had died, and all except 1 patient had an ACCI score of 4 or more (Supplementary Table S5).

## Discussion

In this study, we investigated the impact of comorbidity burden on mortality in patients with COVID-19 from a nationwide claims database. The main finding of our study was that comorbid hypertension, diabetes, congestive heart failure, chronic pulmonary disease, liver disease, renal disease, dementia, and cancer were identified as significant risk factors for mortality in patients with COVID-19 after age and sex adjustment. The predictive performance of the ACCI was superior to that of the CCI. ACCI > 3.5 was found to be the optimal cut-off value for the prediction of death in patients with COVID-19. Our results can provide useful prognostic information to health care professionals, allowing the selection of patients in most need of medical attention and resources.

The mortality rate reported in our study was lower than that reported in studies conducted in other countries^5,12^. According to the recent reports from WHO and the Korea Centers for Disease Control and Prevention (KCDC) (last updated June 2, 2020), mortality rates in Europe, Americas, and South-East Asia were 8.4, 5.7, and 2.8%, respectively. These differences may be explained by several factors. One possible explanation for the difference in mortality rates between is the different clinical characteristics of the populations. The risk of COVID-19 mortality has been consistently reported to increase in male patients, patients with an advanced age, and patients with comorbidities, similar to the observations in our study. The patients in our study were relatively younger and had fewer comorbidities than those reported in studies from other countries^5,12^. These characteristics might be associated with the early COVID-19 outbreak in a relatively large number of young people in South Korea^13,14^, striking differences between Asian and European mortality might indicate the effect of ethnicity on disease outcome^15^. However, because ethnicity is a complex entity composed of social constructs, cultural identity, genetic make-up, and behavioral patterns^16^, it might be difficult to conclude the association between ethnicity and disease outcome. In addition, differences in the organization of health care systems and strategies to contain COVID-19 among different countries may have affected the result. Korea’s rapid and extensive diagnostic testing (more than 10,000 tests daily), and intensive anti-contagion policies may have contributed to the disease outcome^17^.

In this study, we adjusted both sex and age that could affect the prevalence of comorbidities to investigate its effects on the severity of COVID-19. Further, some studies have suggested that male sex is a risk factor for the severity of COVID-19^18,19^. It has been suggested that the sex-based difference between the circulating angiotensin-converting enzyme (ACE)-2 levels, the receptor of which was associated with intracellular penetration of SARS-CoV-2^20^, or the smoking rate difference according to sex may have affected the sex difference on the severity of COVID-19^19,21^. In addition, the potential association between androgen level and COVID-19 severity was suggested^22^. Our results also revealed the tendency toward sex difference in the mortality of COVID-19. Further research is needed to assess the effect of sex on the severity of COVID-19. Age has consistently been reported to affect the severity of COVID-19 in several studies^23,24^. In addition to the increased prevalence of comorbidities in older patients^25^, physiological changes caused by aging itself may affect the severity of COVID-19. Aging leads to the impaired functioning of various systems, including the immune system, resulting in a greater susceptibility to inflammation or death^26,27^. Frailty, which is commonly associated with the elderly, can also affect the prognosis of COVID-19^28^. Therefore, we adjusted age and sex in each analysis to investigate the effects of various comorbidities on patient mortality due to COVID-19.

The results from our study revealed multiple risk factors that were associated with mortality in patients with COVID-19 after age and sex adjustment. SARS-CoV-2 binds to the target cells through the ACE-2 receptor expressed in epithelial cells of several organs^20^. Because the expression of ACE-2 is increased in patients with hypertension, diabetes, and chronic obstructive pulmonary disease (COPD), these comorbidities can increase both the risk and severity of COVID-19 infection^29,30^. In addition, evidence of myocardial or liver damage has been observed in patients with COVID-19, and pre-existing cardiovascular and liver diseases could be associated with the severity of COVID-19 infection^31,32^. Recent meta-analyses have identified that cardiovascular diseases and COPD can greatly affect the severity of COVID-19^33,34^. Renal disease, dementia, and cancer could also be important risk factors for severe COVID-19^35–37^. An association between these comorbidities and the severity of COVID-19 as defined by other indicators (oxygen therapy, mechanical ventilation, ECMO, and cardiopulmonary resuscitation) has been reported in a recent study from the OpenData4Covid19^38^. The effects of each comorbidity on the COVID-19 mortality have been observed in our results as well, and if they are combined, the effect will be stronger on the severity of COVID-19.

Our study showed that the ACCI could be more useful in predicting the mortality of patients with COVID-19, compared to the CCI. A study of 52 critically ill patients with COVID-19 revealed that the median duration between the onset of symptoms and intensive care unit admission was 9–10 days, suggesting a gradual progression of the disease^39^. Therefore, the early detection of risk factors that can predict the severity of disease can improve the patient's prognosis and enable an efficient allocation of medical resources. To this end, we have created a simple but powerful prediction model for the mortality of COVID-19, combining age and comorbidities, known to be important risk factors for the severity of COVID-19. In addition, the high predictive power of the ACCI for mortality in our results could support the importance of old age and comorbidities in the severity of COVID-19. To date, several prognostic models for the severity of COVID-19 have been suggested^40,41^. The predictive value of ACCI in our study was similar to the recently reported clinical risk score that predicts the occurrence of critical illness in hospitalized patients with COVID-19 (development cohort: area under the receiver operating characteristic [AUROC] 0.88 [95% CI 0.85–0.91], validation cohort: AUROC 0.88 [95% CI 0.84–0.93])^40^. Therefore, our findings could be useful for healthcare policy-making on the allocation of limited medical resources in the COVID-19 pandemic^42,43^.

The results of our study should be interpreted cautiously for several reasons. First, the data from insurance claims did not contain detailed clinical information such as vital signs or laboratory values. Although the ACCI showed an excellent predictive value without them, they would have provided other important information regarding the prognosis of COVID-19. Second, the CCI does not consider the use of drugs and relies on diagnosis codes only; thus, over- or underestimation of the risk is likely to have happened. Third, due to the different medical situations and resources for the COVID-19 crisis in each country, the generalizability of the results may be limited. However, the contribution of this study is that it uses nationwide data to provide predictions on the risk of mortality, which is the most serious outcome of COVID-19 infection. Finally, the original ACCI did not include hypertension, which is the most common comorbidity in patients with COVID-19. The development of a COVID-19-specific comorbidity scoring system will be necessary.

In conclusion, our study identified that the ACCI, combined with age and various comorbidities, was associated with mortality in patients with COVID-19 in South Korea. If an increasing number of patients with COVID-19 develop severe illness, plans should be made at the national level to better manage the surge and ensure the need for critical care resources. Furthermore, because the availability of medical resources for critical care is likely restrictive, resource allocation policies based on risk factors should be implemented by medical professionals and policy makers. We hope that our study findings will provide important information to guide health care professionals, who are facing the global health threat of COVID-19, in timely decision-making.

## Methods

This study was reviewed and approved by the institutional review board (IRB) of Seoul National University Hospital (IRB No. E-2004-165-1119), and the requirement of informed consent was waived because the data did not contain any identifiable information. All methods were performed in accordance with the approved guidelines and regulations^44^.

This retrospective cohort study analyzed data from the health insurance claims relevant to COVID-19, submitted to the Health Insurance Review and Assessment (HIRA) of South Korea by May 15, 2020. The HIRA currently provides the data on its website under the project OpenData4Covid19 (https://hira-covid19.net/)^3^. The current dataset includes a total of 234,427 patients who visited the hospital for the diagnosis of COVID-19 and their health insurance claims between January 1, 2017 and May 15, 2020. The dataset was merged with the COVID-19 confirmation and mortality data from KCDC. Given the obligatory nature of the National Health Insurance system (NHIS), our data covers virtually all Koreans (about 50 million people) and captures the clinical data from all healthcare institutions, i.e., clinics, pharmacies, and hospitals^45^. Each healthcare institution must submit all patients’ information regarding diagnosis, treatment, medical services rendered, and drug prescriptions to the NHIS, to receive reimbursement^46^.

In South Korea, COVID-19 diagnostic tests, SARS-CoV-2 real-time polymerase chain reaction is mostly used for persons who have been in contact with COVID-19-positive patients or persons with symptoms that are suspicious of COVID-19^47,48^. We defined the confirmation of COVID-19 using the confirmation code provided by the HIRA, based on the KCDC data^3^. Information on the demographics of participants, type of insurance (health insurance and medical aid), and comorbidities was based on claims codes. Patients with comorbidities were defined as those who had more than three claim records between January 2019 and before COVID-19 testing. This was done to avoid over-estimating the comorbidities and to include only those comorbidities that could probably affect the patient’s recent medical condition. The ACCI values were drawn from the claims data using the International Statistical Classification of Diseases and Related Health Problems, 10th edition (ICD-10) coding algorithm proposed by Quan et al.^49^. The algorithm was applied and validated for the national health insurance claims data in South Korea^50,51^. The ACCI is a weighted measure that incorporates age into the original CCI^11^. In the ACCI, an additional point is added for each decade after 40 years of age (from 1 point for the age group 50–59 years to 4 points for the age group greater than 80 years old). Primary hypertension (ICD-10 code I10.x), which is not included in the CCI, was also identified by the aforementioned method.

The main outcome of our study was patient death due to COVID-19. We did not investigate the effects of the comorbidities on the infection rate or the hospitalization rate of COVID-19. We thought that both medical and non-medical conditions, such as the capacity of the medical resources or social factors in the area, could have affected the infection rate or hospitalization rate of COVID-19. For example, the mass outbreaks in some specific groups in South Korea occurred mainly in young age groups with active social activities^13,52^. These occurrences may result in bias on the medical conditions of the patients with COVID-19.

We performed subgroup analysis for patients with COVID-19 in the Daegu and Gyeongbuk regions with a population of about five million, where most confirmed cases of COVID-19 were reported in South Korea. In South Korea, there has been a surge in the number of confirmed COVID-19 cases in a religious sect gathering called the Shincheonji^53^. This rapid spread has led to a shortage of hospital beds and healthcare professionals in this area^54^. We thought that the situation in this region better reflected the current crisis in other countries with a high COVID-19 prevalence. In addition, we performed a comparative analysis between male and female patients to investigate the effect of sex on COVID-19 mortality.

### Statistical analysis

The baseline characteristics are presented as mean ± standard deviation for continuous variables and as frequencies with percentages for categorical variables. Continuous variables were compared using unpaired Student’s *t*-test, and categorical variables were compared using either the chi-square test or Fisher’s exact test, as appropriate. We calculated the age- and sex-adjusted ORs of mortality by comorbidities included in the CCI, using the logistic regression model. For age adjustment, age was classified into ten-yearly age groups (0–9, 10–19, 20–29, 30–39, …years) as a categorical variable. We also performed a multivariable logistic regression analysis with the CCI score as the covariate. The ability of the CCI and ACCI to predict in-hospital mortality among all patients with COVID-19 or hospitalized patients with COVID-19 was determined using the ROC curves and their AUROC. To evaluate the predicting performance, we calculated the AUROC, sensitivity, specificity, PPV, and NPV. DeLong's test was used to compare the ROC curves. All statistical tests were performed with SAS, version 9.4 (SAS Institute; Cary, NC), or R software version 3.6.3 (R Core Team, 2020. R: A language and environment for statistical computing. R Foundation for Statistical Computing, Vienna, Austria. https://www.R-project.org/).

## Supplementary Information

Supplementary Information.
