# Supplementary material for: Impact of comorbidity burden on mortality in patients with COVID-19 using the Korean health insurance database
Source: Sci Rep. 2021 Mar 18;11:6375. doi: 10.1038/s41598-021-85813-2 (PMC7973767; doi:10.1038/s41598-021-85813-2)

**Impact of comorbidity burden on mortality in patients with COVID-19 using the Korean health insurance database**

Soo Ick Cho^1#^, Susie Yoon^2,3#^, and Ho-Jin Lee^2,3*^

**From**

^1^ Department of Dermatology, Seoul National University Hospital, Seoul, Republic of Korea

^2^ Department of Anesthesiology and Pain Medicine, Seoul National University Hospital, Seoul, Republic of Korea

^3^ Department of Anesthesiology and Pain Medicine, Seoul National University College of Medicine, Seoul, Republic of Korea

^#^ These two authors equally contributed to this work as co-first authors.

***Corresponding Author:** Ho-Jin Lee, MD

Address: Department of Anesthesiology and Pain Medicine, Seoul National University Hospital, 101 Daehak-ro, Jongno-gu, Seoul 03080, Republic of Korea

Phone: 82-2-2072-2467

FAX: 82-2-747-8363

E-mail: zenerdiode03@gmail.com

**Supplemental Table S1**. Baseline characteristics and comorbidity of patients with coronavirus disease (COVID-19) according to sex.

|  | Male (n=3,095) | Female (n=4,495) | P-value |
| --- | --- | --- | --- |
| Death | 121 (3.9) | 106 (2.4) | < 0.001 |
| Age, years |  |  | < 0.001 |
| -9 | 46 (1.5) | 36 (0.8) |  |
| 10-19 | 180 (5.8) | 166 (3.7) |  |
| 20-29 | 826 (26.7) | 1029 (22.9) |  |
| 30-39 | 362 (11.7) | 414 (9.2) |  |
| 40-49 | 330 (10.7) | 673 (15.0) |  |
| 50-59 | 527 (17.0) | 976 (21.7) |  |
| 60-69 | 444 (14.3) | 617 (13.7) |  |
| 70-79 | 258 (8.3) | 332 (7.4) |  |
| 80- | 122 (3.9) | 252 (5.6) |  |
| Insurance type |  |  | 0.005 |
| Health insurance | 2804 (90.6) | 4154 (92.4) |  |
| Medical aid | 291 (9.4) | 341 (7.6) |  |
| Cities and province |  |  | 0.359 |
| Daegu-Gyeongbuk region | 1707 (55.2) | 2527 (56.2) |  |
| Other cities and provinces | 1388 (44.8) | 1968 (43.8) |  |
| Comorbidity |  |  |  |
| Hypertension | 625 (20.2) | 838 (18.6) | 0.092 |
| Diabetes mellitus | 436 (14.1) | 471 (10.5) | < 0.001 |
| Congestive heart failure | 91 (2.9) | 119 (2.6) | 0.445 |
| Cerebrovascular disease | 172 (5.6) | 220 (4.9) | 0.200 |
| Liver disease | 270 (8.7) | 338 (7.5) | 0.058 |
| Renal disease | 31 (1.0) | 28 (0.6) | 0.065 |
| Chronic pulmonary disease | 356 (11.5) | 602 (13.4) | 0.015 |
| Cancer | 95 (3.1) | 168 (3.7) | 0.118 |

Data are presented as the number (%).

**Supplemental Table S2**. Results of multivariable logistic regression analysis for factors associated with mortality in patients with COVID-19.

|  |  | Unadjusted OR  (95% CI) | P-value | Adjusted OR^a^  (95% CI) | P-value | Adjusted OR^b^  (95% CI) | P-value |
| --- | --- | --- | --- | --- | --- | --- | --- |
| CCI score (vs. 0) | 1 | 4.61 (2.69-7.88) | <0.001 | 1.46 (0.83-2.56) | 0.191 | 1.41 (0.80-2.48) | 0.237 |
|  | 2 | 12.07 (7.29-19.96) | <0.001 | 2.04 (1.19-3.51) | 0.010 | 1.89 (1.09-3.27) | 0.023 |
|  | 3 | 21.79 (13.11-36.22) | <0.001 | 2.96 (1.71-5.14) | <0.001 | 2.71 (1.55-4.73) | <0.001 |
|  | 4 | 57.25 (34.31-95.53) | <0.001 | 5.69 (1.71-5.14) | <0.001 | 5.08 (2.85-9.06) | <0.001 |
|  | 5 | 64.81 (36.61-114.73) | <0.001 | 7.31 (3.91-13.67) | <0.001 | 6.51 (3.44-12.31) | <0.001 |
|  | 6 | 37.99 (16.22-89.01) | <0.001 | 3.68 (1.46-9.30) | 0.006 | 3.20 (1.26-8.17) | 0.015 |
|  | 7 | 49.25 (19.70-123.12) | <0.001 | 5.78 (2.09-16.00) | <0.001 | 5.15 (1.86-14.29) | 0.002 |
|  | 8 | 75.99 (22.39-257.86) | <0.001 | 11.84 (2.88-48.77) | <0.001 | 10.68 (2.56-44.52) | 0.001 |
|  | 9 | 113.98 (25.89-501.86) | <0.001 | 19.47 (3.96-95.68) | <0.001 | 16.54 (3.34-81.90) | <0.001 |
| Age (per 10 years) |  |  |  | 2.88 (2.48-3.35) | <0.001 | 2.80 (2.40-3.27) | <0.001 |
| Female (vs. male) |  |  |  | 0.46 (0.34-0.63) | <0.001 | 0.46 (0.34-0.63) | <0.001 |
| Daegu-Gyeongbuk region |  |  |  | 2.20 (1.42-3.43) | <0.001 | 2.20 (1.42-3.42) | <0.001 |
| Hypertension |  |  |  |  |  | 1.38 (0.99-1.92) | 0.062 |

CCI, Charlson comorbidity index; CI, confidence interval; OR, odds ratio

^a^ This model was adjusted for age, sex, and region (Daegu-Gyeonbuk region vs other regions).

^b^ This model was adjusted for age, sex, region (Daegu-Gyeonbuk region vs other regions), and hypertension.

**Supplemental Table S3**. Sensitivity and specificity of the Charlson comorbidity index (CCI) in predicting mortality among patients with coronavirus disease (COVID-19) according to sex.

|  | Sensitivity | Specificity | Youden index | PPV | NPV |
| --- | --- | --- | --- | --- | --- |
| Male |  |  |  |  |  |
| Age-adjusted |  |  |  |  |  |
| CCI ≥ 2 | 0.975 | 0.664 | 1.639 | 0.106 | 0.998 |
| CCI ≥ 3 | 0.917 | 0.765 | 1.683 | 0.137 | 0.996 |
| CCI ≥ 4 | 0.835 | 0.841 | 1.676 | 0.176 | 0.992 |
| Unadjusted |  |  |  |  |  |
| CCI ≥ 1 | 0.860 | 0.684 | 1.543 | 0.100 | 0.992 |
| CCI ≥ 2 | 0.744 | 0.823 | 1.567 | 0.146 | 0.987 |
| CCI ≥ 3 | 0.595 | 0.906 | 1.501 | 0.205 | 0.982 |
| Female |  |  |  |  |  |
| Age-adjusted |  |  |  |  |  |
| CCI ≥ 3 | 0.953 | 0.762 | 1.715 | 0.088 | 0.999 |
| CCI ≥ 4 | 0.906 | 0.841 | 1.747 | 0.121 | 0.997 |
| CCI ≥ 5 | 0.830 | 0.898 | 1.729 | 0.165 | 0.995 |
| Unadjusted |  |  |  |  |  |
| CCI ≥ 1 | 0.915 | 0.662 | 1.577 | 0.061 | 0.997 |
| CCI ≥ 2 | 0.783 | 0.831 | 1.614 | 0.101 | 0.994 |
| CCI ≥ 3 | 0.585 | 0.915 | 1.499 | 0.142 | 0.989 |

PPV, positive predictive value; NPV, negative predictive value

**Supplemental Table S4**. Distributions of the Charlson comorbidity index (CCI) and age-adjusted CCI (ACCI) in the all-patients and the Daegu-Gyeongbuk cohort.

|  |  | CCI score | | | | | | | | |
| --- | --- | --- | --- | --- | --- | --- | --- | --- | --- | --- |
|  |  | 0 | 1 | 2 | 3 | 4 | 5 | 6 | 7 | ≥8 |
| Total | CCI |  |  |  |  |  |  |  |  |  |
|  | Survival  (n = 7,363) | 4,939 (67.1) | 1,155 (15.7) | 614  (8.3) | 340  (4.6) | 146  (2.0) | 85  (1.2) | 40  (0.5) | 27  (0.4) | 17  (0.2) |
|  | Non-survival  (n = 227) | 26  (11.5) | 28  (12.3) | 39  (17.2) | 39  (17.2) | 44  (19.4) | 29  (12.8) | 8  (3.5) | 7  (3.1) | 7  (3.1) |
|  | ACCI |  |  |  |  |  |  |  |  |  |
|  | Survival  (n = 7,363) | 3,447  (46.8) | 1,339  (18.2) | 834  (11.3) | 573  (7.8) | 408  (5.5) | 296  (4.0) | 205  (2.8) | 117  (1.6) | 144  (2.0) |
|  | Non-survival  (n = 227) | 2  (0.9) | 4  (1.8) | 9  (4.0) | 15  (6.6) | 28  (12.3) | 21  (9.3) | 40  (17.6) | 39  (17.2) | 69  (30.4) |
| Daegu-Gyeongbuk | CCI |  |  |  |  |  |  |  |  |  |
|  | Survival  (n = 4,033) | 2,395  (59.4) | 713  (17.7) | 420  (10.4) | 250  (6.2) | 114  (2.8) | 74  (1.8) | 29  (0.7) | 24  (0.6) | 14  (0.3) |
|  | Non-survival  (n = 201) | 21  (10.4) | 24  (11.9) | 35  (17.4) | 35  (17.4) | 41  (20.4) | 28  (13.9) | 7  (3.5) | 5  (2.5) | 5  (2.5) |
|  | ACCI |  |  |  |  |  |  |  |  |  |
|  | Survival  (n = 4,033) | 1,518  (37.6) | 713  (17.7) | 529  (13.1) | 393  (9.7) | 295  (7.3) | 200  (5.0) | 165  (4.1) | 96  (2.4) | 124  (3.1) |
|  | Non-survival  (n = 201) | 2  (1.0) | 4  (2.0) | 6  (3.0) | 9  (4.5) | 28  (13.9) | 20  (10.0) | 34  (16.9) | 36  (17.9) | 62  (30.8) |

**Supplemental Table S5**. Distributions of the Charlson comorbidity index (CCI) and age-adjusted CCI (ACCI) in the non-hospitalized patients

|  |  | CCI score | | | | | | | | |
| --- | --- | --- | --- | --- | --- | --- | --- | --- | --- | --- |
|  |  | 0 | 1 | 2 | 3 | 4 | 5 | 6 | 7 | ≥8 |
| Total  (n = 433) | CCI |  |  |  |  |  |  |  |  |  |
|  | Survival  (n = 424) | 303  (71.5) | 60  (14.2) | 32  (7.5) | 17  (4.0) | 6  (1.4) | 3  (0.7) | 2  (0.5) | 0  (0.0) | 1  (0.2) |
|  | Non-survival  (n = 9) | 0  (0.0) | 3  (33.3) | 2  (22.2) | 0  (0.0) | 1  (11.1) | 2  (22.2) | 1  (11.1) | 0  (0.0) | 0  (0.0) |
|  | ACCI |  |  |  |  |  |  |  |  |  |
|  | Survival  (n = 424) | 215  (50.7) | 80  (18.9) | 45  (10.6) | 29  (6.8) | 26  (6.1) | 11  (2.6) | 13  (3.1) | 1  (0.2) | 4  (0.9) |
|  | Non-survival  (n = 9) | 0  (0.0) | 0  (0.0) | 0  (0.0) | 1  (11.1) | 4  (44.4) | 0  (0.0) | 0  (0.0) | 1  (11.1) | 3  (33.3) |
| Daegu-Gyeongbuk  (n = 347) | CCI |  |  |  |  |  |  |  |  |  |
|  | Survival  (n = 338) | 238  (70.4) | 44  (13.0) | 30  (8.9) | 14  (4.1) | 6  (1.8) | 3  (0.9) | 2  (0.6) | 0  (0.0) | 1  (0.3) |
|  | Non-survival  (n = 9) | 0  (0.0) | 3  (33.3) | 2  (22.2) | 0  (0.0) | 1  (11.1) | 2  (22.2) | 1  (11.1) | 0  (0.0) | 0  (0.0) |
|  | ACCI |  |  |  |  |  |  |  |  |  |
|  | Survival  (n = 338) | 170  (50.3) | 58  (17.2) | 35  (10.4) | 25  (7.4) | 24  (7.1) | 10  (3.0) | 11  (3.3) | 1  (0.3) | 4  (1.2) |
|  | Non-survival  (n = 9) | 0  (0.0) | 0  (0.0) | 0  (0.0) | 1  (11.1) | 4  (44.4) | 0  (0.0) | 0  (0.0) | 1  (11.1) | 3  (33.3) |

**Supplemental Figure 1.** Age- and sex-adjusted odds ratios (95% confidence interval [CI]) of mortality according to the comorbidity of male patients (red) and female patients (blue) with coronavirus disease (COVID-19). CHF, chronic heart failure; CPD, chronic pulmonary disease; CVD, cerebrovascular disease; DM, diabetes mellitus; HTN, hypertension; MI, myocardial infarction; NA, not available. R: A language and environment for statistical computing. R Foundation for Statistical Computing, Vienna, Austria. https://www.R-project.org/.


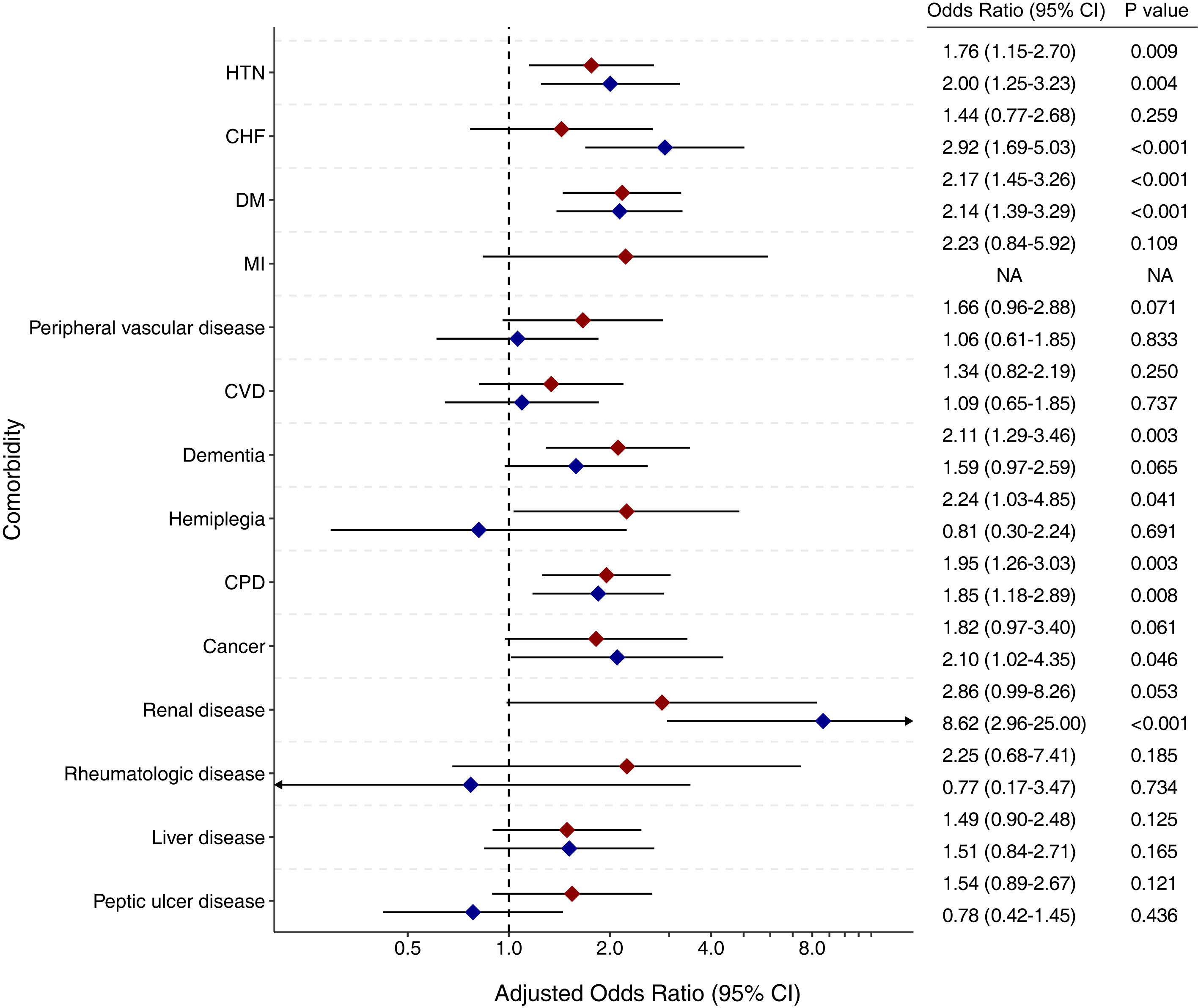


**Supplemental Figure 2.** The area under the receiver operative characteristic curves of the Charlson comorbidity index (CCI) for predicting mortality of male patients (A) and female patients (B) with COVID-19. The blue and red lines represent unadjusted and age-adjusted CCI, respectively. ACCI, age-adjusted Charlson comorbidity index; AUROC, area under the receiver operating characteristic. R: A language and environment for statistical computing. R Foundation for Statistical Computing, Vienna, Austria. https://www.R-project.org/.


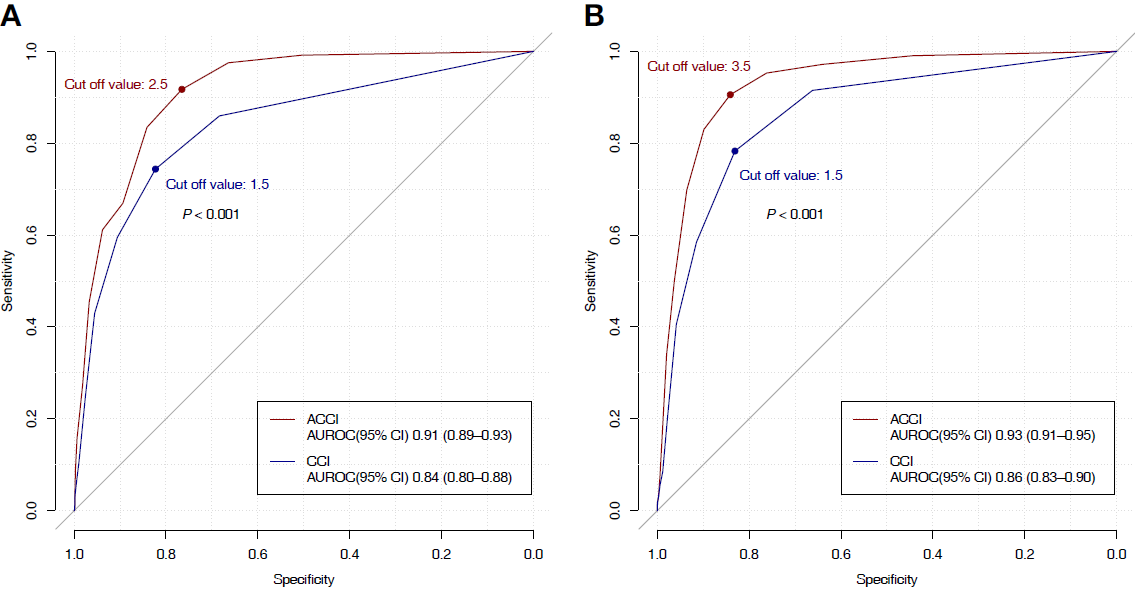

Supplement: Supplementary file 1 — Supplementary Information. [file 41598_2021_85813_MOESM1_ESM.docx]
